# hsa-mir-30c promotes the invasive phenotype of metastatic breast cancer cells by targeting NOV/CCN3

**DOI:** 10.1186/s12935-014-0073-0

**Published:** 2014-08-02

**Authors:** Jason R Dobson, Hanna Taipaleenmäki, Yu-Jie Hu, Deli Hong, Andre J van Wijnen, Janet L Stein, Gary S Stein, Jane B Lian, Jitesh Pratap

**Affiliations:** 1 Department of Cell and Developmental Biology, University of Massachusetts Medical School, 55 Lake Ave, North, Worcester 01655, MA, USA; 2 Current address: Center for Computational Molecular Biology, Department of Molecular Biology, Cell Biology, and Biochemistry, and Department of Computer Science, Brown University, 115 Waterman Street, Providence 02912, RI, USA; 3 Current address: Heisenberg-Group for Molecular Skeletal Biology, Department of Trauma, Hand, and Reconstructive Surgery, University Medical Center Hamburg-Eppendorf, Hamburg, Germany; 4 Current address: Department of Biochemistry and Vermont Cancer Center, University of Vermont College of Medicine, 89 Beaumont Avenue, Burlington 05405-0068, VT, USA; 5 Current address: Departments of Orthopedic Surgery and Biochemistry & Molecular Biology, Mayo Clinic, 200 First Street SW, Medical Sciences Building 3-69, Rochester 55905, MN, USA; 6 Current address: Department of Anatomy and Cell Biology, Rush University Medical Center, Armour Academic Center, 600 S, Paulina Street, Suite 507, Chicago 60612, IL, USA

**Keywords:** miRNA, Hsa-mir-30c breast cancer, Invasion, Metastasis, NOV/CCN3

## Abstract

**Background:**

For treatment and prevention of metastatic disease, one of the premier challenges is the identification of pathways and proteins to target for clinical intervention. Micro RNAs (miRNAs) are short, non-coding RNAs, which regulate cellular activities by either mRNA degradation or translational inhibition. Our studies focused on the invasive properties of hsa-mir30c based on its high expression in MDA-MB-231 metastatic cells and our bioinformatic analysis of the Cancer Genome Atlas that identified aberrant hsa-mir-30c to be associated with poor survival.

**Methods:**

Contributions of hsa-mir-30c to breast cancer cell invasion were examined by Matrigel invasion transwell assays following modulation of hsa-mir-30c or hsa-mir-30c* levels in MDA-MB-231 cells. hsa-mir-30c *in silico* predicted targets linked to cell invasion were screened for targeting by hsa-mir-30c in metastatic breast cancer cells by RT-qPCR. The contribution to invasion by a target of hsa-mir-30c, Nephroblastoma overexpressed (NOV), was characterized by siRNA and invasion assays. Significant effects were determined using Student’s T-tests with Welch’s correction for unequal variance.

**Results:**

MCF-7 and MDA-MB-231 cells were used as models of poorly invasive and late-stage metastatic disease, respectively. By modulating the levels of hsa-mir-30c in these cells, we observed concomitant changes in breast cancer cell invasiveness. From predicted targets of hsa-mir-30c that were related to cellular migration and invasion, NOV/CCN3 was identified as a novel target of hsa-mir-30c. Depleting NOV by siRNA caused a significant increase in the invasiveness of MDA-MB-231 cells is a regulatory protein associated with the extracellular matrix.

**Conclusions:**

NOV/CCN3 expression, which protects cells from invasion, is known in patient tumors to inversely correlate with advanced breast cancer and metastasis. This study has identified a novel target of hsa-mir-30c, NOV, which is an inhibitor of the invasiveness of metastatic breast cancer cells. Thus, hsa-mir-30c-mediated inhibition of NOV levels promotes the invasive phenotype of MDA-MB-231 cells and significantly, the miR-30/NOV pathways is independent of RUNX2, a known target of hsa-mir-30c that promotes osteolytic disease in metastatic breast cancer cells. Our findings allow for mechanistic insight into the clinical observation of poor survival of patients with elevated hsa-mir-30c levels, which can be considered for miRNA-based translational studies.

## Background

Breast cancer is the most commonly diagnosed disease among women. Aggressive breast cancers have high potential to become metastatic, a transition that makes clinical intervention difficult. In recent years, many of the molecular mechanisms that facilitate a more invasive or metastatic state have been characterized [1]–[3]. It has been observed that the transcriptome of primary breast cancer cells with a predisposition for metastasis can be distinguished from non-metastatic breast cancer cells [4]. This indicates that steady-state mRNA levels are altered in pre-metastatic tissue to promote metastasis. Therefore, it is critical to define the events leading to and maintaining a metastatic transcriptional landscape of metastatic breast cancer cells.

Assessing a cell’s transcriptome using RNA-seq or hybridization arrays provides measurement of the steady-state levels of cellular mRNAs. Cellular mRNA levels are controlled at multiple levels: transcriptional rate; mRNA processing and export; mRNA stability; and translational rate. miRNAs are small non-coding RNAs that post-transcriptionally regulate mRNA levels, many of which are critical for development [5]. Similar to mRNAs, the steady-state levels of miRNAs are also altered in cancerous tissues as compared to normal cells. Further, just as for protein coding RNAs, many non-coding RNAs have the capacity to function as tumor suppressors and oncogenes (often referred to as onco-miRs) [6],[7]. miRNAs are also involved in cancer cell metastasis (metasta-miRs), and regulate key physiological steps in the metastatic process of many cancers, including breast [2],[8]–[11]. Hence, characterizing members of this class of non-coding RNAs is important in our understanding of breast cancer development and progression.

We queried publicly available data from TCGA Breast Cancer project [12] for aberrant miRNAs, and observed that hsa-mir-30c is elevated in expression level or copy number in approximately 4% or breast cancer patients. Importantly, these patients have significantly poorer survival than patients with normal hsa-mir-30c, which suggests that hsa-mir-30c may be a key factor in breast cancer progression. Using a cell-based model, we observed an association between cellular invasion and high levels hsa-mir-30c, which led us to hypothesize that the observed clinical mortality phenotype associated with aberrant hsa-mir-30c was as a result of hsa-mir-30c regulation of breast cancer cell invasion. To address this hypothesis, we examined factors targeted by hsa-mir-30c that regulate breast cancer cell invasion. We investigated potential cross talk between a factor known to be targeted by miR-30c, RUNX2 [13], which promotes metastasis and osteolytic disease [14]. Further, because there are still many unknown players promoting the metastatic phenotype of advanced breast cancer, we addressed other targets of hsa-mir-30c, first by *in silico* analyses for targets linked to cellular invasion, then functional investigation of these potential targets.

Our key findings demonstrated that highly metastatic MDA-MB-231 breast cancer cells have robust levels of hsa-mir-30c compared to non-metastatic MCF-7 cells; and that hsa-mir-30c promotes breast cancer cellular invasion through targeting of NOV/CCN3, which we characterized as an inhibitor of invasion. We demonstrated the specificity of this pathway by showing: a) that only the canonical strand of hsa-mir-30c is detected and responsible for the invasive phenotype; and b) that hsa-mir-30c-NOV/CCN3-mediated invasiveness is completely independent of hsa-mir-30c targeting of RUNX2. Importantly, our cell-based experimental observations allow for mechanistic insight into the clinical observations of both hsa-mir-30c and NOV/CCN3, which suggests that the hsa-mir-30c-NOV pathway is an important target for future translational studies.

## Results

### hsa-mir-30c promotes the invasiveness of MDA-MB-231 breast cancer cells

**Figure 1:**
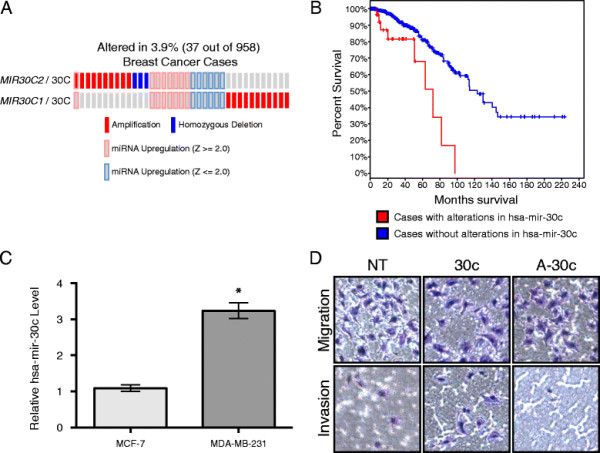
**hsa-mir-30c promotes the invasiveness of MDA-MB-231 cells and regulates RUNX2 levels. (A)** Oncoprint of breast cancer samples that have altered copy number in *MIR30C1* or *MIR30C2*, or significantly differentially expressed levels of the mature miRNA, hsa-mir-30c. Differentially expressed samples are defined by having a Z score greater than 2.0 or less than −2.0. Z is calculated as the difference between the expression value of hsa-mir-30c and the mean expression value of hsa-mir-30c in samples that are diploid for *MIR30C1/MIR30C2* divided by the standard deviation of the expression values for hsa-mir-30c in samples that are diploid for *MIR30C1/MIR30C2*. **(B)** Survival plot of patients with copy number alterations in *MIR30C1/MIR30C2* or levels of hsa-mir-30c (red) versus patients with diploid *MIR30C1/MIR30C2* or normal levels of hsa-mir-30c. Log-rank *P*-value < 1.09e-03. **(C)** Real Time qPCR detection of hsa-mir-30c in MCF-7 and MDA-MB-231 cells, normalized to U6 RNA. Mean and SEM (error bars) for two technical replicates of two biological replicates. * = Student’s t-test with Welch correction p-value < 0.05. **(D)** Representative Matrigel invasion assay using MDA-MB-231 cells following 48 h of transient transfection with non-targeting miRNA (NT), hsa-mir-30c (30c) or anti-hsa-mir-30c (A-30c); cells were stained with HEMA3.

Numerous miRNAs have been implicated in tumorigenesis, driving tumor progression, or promoting metastases; however, there are still many miRNAs that have yet to be characterized with respect to these oncogenic processes. Using cBioPortal [15],[16] to investigate the genomics and transcriptomics of TCGA breast cancer patients [12], we observed frequent amplifications in the genes *MIR30C1* and *MIR30C2*, which encode the same mature miRNA, hsa-mir-30c, as well as many patients with significant (absolute value of Z-score > =2.0) changes in the levels of hsa-mir-30c (Figure 1A). Importantly, patients with alterations in copy number or expression of hsa-mir-30c have significantly poorer (log-rank *P*-value < 1.09e-03) survival than their wild-type counterparts (Figure 1B). Because many of the observed alteration of hsa-mir-30c in patents were amplifications, increased expression levels, or both we sought to use cell-based models to characterize the functional impact of modulation of hsa-mir-30c.

*In vitro*, MCF-7 breast cancer cells have been observed to be less invasive than MDA-MB-231 cells [17]. Using Real Time qPCR, we observed statistically significantly higher levels of endogenous hsa-mir-30c in the more invasive breast cancer cell line MDA-MB-231 compared to the MCF7 cell line (Figure 1C). This finding suggests that increased expression of hsa-mir-30c is associated with more invasive breast cancer cells. To address the effect of hsa-mir-30c on the invasive potential of MDA-MB-231 cells, MDA-MB-231 cells were transfected with either hsa-mir-30c or anti-mir-30c for 48 h and loaded the cells into the top of the transwell culture plates with or without a layer of Matrigel. Conditioned osteoblast media was placed in the bottom well as a chemoattractant for the cells to mimic a bone environment (Figure 1D). Using this assay, we observed that more MDA-MB-231 cells transfected with hsa-mir-30c invaded through the Matrigel as compared to control transfected MDA-MB-231 cells, which indicates that hsa-mir-30c promotes the invasiveness of MDA-MB-231 cells. When MDA-MB-231 cells were transfected with anti-mir-30c, we observed that less cells had invaded through the Matrigel as compared to control transfected MDA-MB-231 cells. Taken together, we conclude that hsa-mir-30c expression levels are linked with the invasive potential of MDA-MB-231 metastatic breast cancer cells.

### The canonical 5-prime end of hsa-mir-30c, not the “star” or 3-prime end of hsa-mir-30c, promotes the invasiveness of MDA-MB-231 cells

During normal miRNA biogenesis, the stem-loop structure that is termed the pre-miRNA is cleaved and only one end of the longer pre-miRNA is integrated into the Dicer complex [18]. In the case of hsa-mir-30c, the 5’-end of the stem-loop is most commonly detected as the mature form of the miRNA [19]. When the “star” strand (or in the case of hsa-mir-30c the 3’-end of the stem-loop) is incorporated into the Dicer complex, a unique set of mRNAs is predicted to be targeted for translational repression of mRNA degradation. The phenomenon of alternate utilization of the non-canonical part of the pre-miRNA has been observed in leukemic cells [20]; therefore, we investigated the extent to which this kind of transformation may be occurring in MDA-MB-231 metastatic breast cancer cells.

**Figure 2:**
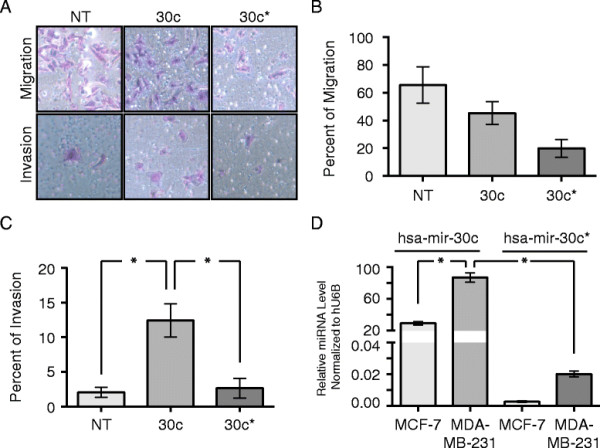
**The 5’-end of the hsa-mir-30c hairpin is the predominant mature miRNA detected in and promoting the invasiveness of MDA-MB-231 cells. (A)** Representative images from Matrigel invasion assay following 48 h of transient transfection of siRNA of cells stained with HEMA3. **(B)** Percent of cells that migrated through the control inserts, defined by count of stained cells on the bottom of the inserts as a percent of cells loaded into inserts. Mean and SEM (error bars) for four technical replicates each of two biological replicates. **(C)** Percent of cells that invaded through the Matrigel inserts, normalized to the number of cells that migrated through the control inserts. Mean with SEM (error bars) for four technical replicates each of two biological replicates. * = Wilcoxon Rank Sum p-value < 0.01. (A-C) NT = non-targeting miRNA, 30c = hsa-mir-30c, and 30c* = hsa-mir-30c-3p. **(D)** qPCR detection of endogenous hsa-mir-30c (hsa-mir-30c-5p) and hsa-mir-30c* (hsa-mir-30c-3p) in MCF-7 and MDA-MB-231 cells. Mean with SEM (error bars) for two technical replicates each of two biological replicates normalized to U6 snRNA using the delta-Ct method. * = p-value from Student’s t-test with Welch correction comparing hsa-mir-30c (hsa-mir-30c-5p) and hsa-mir-30c* (hsa-mir-30c-3p) in MDA-MB-231 cells < 0.05.

To determine whether the hsa-mir-30c-mediated effect on the invasiveness of MDA-MB-231 cells was due to the canonical miRNA strand, we transiently transfected MDA-MB-231 cells with either hsa-mir-30c-5p (canonical) or hsa-mir-30c-3p (star) an measured the invasive properties of MDA-MB-231 cells using a Matrigel transwell assay. Transfection of MDA-MB-231 cells with hsa-mir-30c-3p resulted in a reduction of the number of cells that migrated through the transwells in the absence of Matrigel; however, transfection with hsa-mir-30c-3p did not significantly affect the number of cells that invaded through Matrigel (Figure 2A-C).

We examined endogenous expression of the canonical and star miRNA products of hsa-mir-30c miRNA biogenesis in the breast cancer cell lines (Figure 2D). Using primers specifically designed to detect either the 5’- or 3’-form of hsa-mir-30c, we observed that the ratio of the endogenous levels of the 5’-form were several hundred fold higher than the 3’-form in both MDA-MB-231 and MCF-7 breast cancer cells and that the canonical form is 5 fold higher in the MDA-MB-231 compared to the MCF-7 cells. These results indicate that the phenomenon of “star”-strand miRNA activity of hsa-mir-30c does not make a major contribution to the invasiveness of MDA-MB-231 cells. Instead, only the canonical 5’-strand of hsa-mir-30c appears to be promoting the invasive phenotype of MDA-MB-231 metastatic breast cancer cells.

### Screening via ontological terms and qPCR reveals NOV to be a target of hsa-mir-30c in MDA-MB-231 breast cancer cells

**Figure 3:**
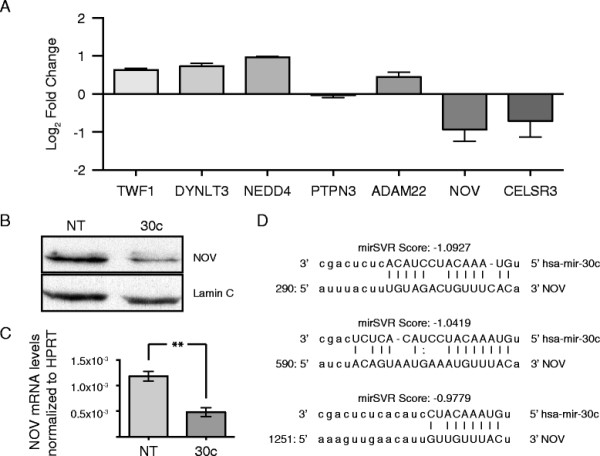
**Predictive, ontological, and qPCR screen for hsa-mir-30c reveals NOV as a target of hsa-mir-30c. (A)** The log_2_ of the fold change in mean with SEM detection levels (hsa-mir-30c/non-targeting miRNA) is plotted for each set of primers for each transcript normalized to HPRT using delta-delta Ct method [51]. **(B)** Representative Western blots for lysates extracted from MDA-MB-231 cells following 48 h of transient transfection with either non-targeting miRNA (NT) or hsa-mir-30c (30c). Top blot: NOV, bottom blot: Lamin C. **(C)** qPCR for NOV levels for two technical replicates each for three biological replicates following 48 h transient transfections of non-targeting miRNA (NT) or hsa-mir-30c (30c) normalized to HPRT using delta-delta Ct method [51]. Mean with SEM. ** = p-value from Student’s t-test with Welch correction < 0.01. **(D)** Alignment of hsa-mir-30c (top sequences) with the 3’-UTR of NOV (bottom sequences) with the 5’-positions within the NOV 3’-UTR being relative to the 5’-start of the 3’-UTR for each of the three predicted targeting sites. Target scores are provided by mirSVR. Uppercase letters linked with a “|” character indicates a perfect match, while uppercase letters linked with a “:” character indicates a wobble pair.

To identify potential targets of hsa-mir-30c participating in the invasive phenotype associated with hsa-mir-30c expression in MDA-MB-231 cells, we performed a screen in three steps: 1) generated a list of potential targets based on seed sequence targeting potential using the top 300 human mRNA targets from www.microrna.org[21]; 2) filtered the list based on known functions and ontological terms for mRNAs that code for proteins associated with invasion, adhesion, or migration, as well as mRNAs that code for transcription factors [22],[23] (Table 1); 3) used qPCR to measure the relative mRNA levels of potential targets in MDA-MB-231 cells that had been transfected with either non-targeting miRNA or hsa-mir-30c. Using this screening approach, we observed that NOV mRNA was one of the two most down regulated *in silico*-identified target upon transfection of hsa-mir-30c (Figure 3A). We also examined several *in silico*-predicted targets related to cancer at the protein level and found these to be unaffected by the levels of hsa-mir-30c (Additional file 1: Figure S1). We focused on NOV, which is a member of the CCN family of matricellular signaling proteins, and has context-specific functions as an oncogene/tumor suppressor [24]–[28]. While CELSR3 had similar response trends to hsa-mir-30c overexpression and is involved in cellular adhesion [29], we did not focus on CELSR3 because the expression levels were very low in MDA-MB-231 cells (data not shown) and CELSR3 was previously shown to not affect invasion in metastatic melanoma cells [30]. In parallel we also looked at *in silico*-predicted targets of hsa-mir-30c that have known functions in cancer and found that none of these predicted targets were affected by overexpression of hsa-mir-30c (Additional file 1: Figure S1). Using Western blotting to detect the levels of NOV protein, we also observed NOV protein to be reduced following transfection of MDA-MB-231 cells with hsa-mir-30c (Figure 3B). The observation that NOV mRNA was reduced upon transfection of hsa-mir-30c was highly reproducible in multiple biological replicates (Figure 3C), consistent with the reduction in protein levels.

**Table 1:** Genes chosen for qPCR screen

**Predicted hsa-mir-30c targets from microRNA.org**		
**Gene symbol**	**mirSVR Score**	**References into function or ontology terms**
TWF1	−3.02	GO: Actin Binding
		Regulates actin filament turnover [31]
		Promotes cellular motility through actin regulation [32]
DYNLT3	−3.02	GO: Cytoplasm, GO: Plasma Membrane
		Cytoplasmic dynein light chain protein that interacts with spindle checkpoint protein BUB3 [33]
		Functions as a nuclear matrix-associated transcription factor in a dynein-independent manner [34]
NEDD4	−2.47	Modulates p-Smad1 signaling in response to both BMP-2 and TGFβ1 [35]
		Negatively regulates PTEN in an oncogenic fashion [36]
		Involved in the deposition of extracellular collagen [37]
PTPN3	−2.55	Regulates EGF-mediated cell-cell contact [38]
		Regulates focal adhesion [39]
		Regulates Cadherin-mediated cell-cell contact [40]
		Regulates neural crest cell adhesion and motility [41]
		Controls hormone receptor signaling [42]
		Cooperates with vitamin D to promote breast cancer cell growth [43]
ADAM22	−2.44	GO: Extracellular, GO: Metallopeptidase Activity
		Metalloproteinase-like, disintegrin-like, cysteine-rich protein that is highly expressed in brain tissue [44]
		Promotes cell adhesion and spreading [45]
		Cooperates with SRC-1 in endocrine resistance of breast cancer cells; expression of ADAM22 independently predicts poor survival in patients [46]
NOV	−3.11	Regulates actin cytoskeletal reorganization [28]
		Increases motility of chondrosarcoma cells [47]
		Promotes mineralization of osteoblasts [48]
		Transcriptionally activated by p53 [24]
		Downregulated in advanced melanomas [26]
		Downregulated in childhood adrenocortical tumors [25]
		Significantly over-expressed in ER-positive breast cancer patients who relapsed after tamoxifen treatment [49]
		Expression is negatively correlated with metastasis and progression in breast cancer [50]
		Promotes xenograph breast cancer bone-metastasis and osteolysis [27]
CELSR3	−4.03	GO: G-protein coupled receptor signaling pathway, GO: neuron migration, and GO: plasma membrane

Genes were chosen for qPCR screen based on: mirSVR score (www.microrna.org) [21], NCBI GeneRIF [22], and ontological terms [23]. TWF1 references: [31],[32]. DYNLT3 references: [33],[34]. NEDD4 references: [35]–[37]. PTPN3 references: [38]–[43]. ADAM22 references: [44]–[46]. NOV references: [24]–[28],[47],[48],[50].

Comparing the sequence of hsa-mir-30c and the sequence of the NOV 3’-UTR, mirSVR [52] predicted three potential binding sites for hsa-mir-30c (Figure 3D). Among the mir-30 family members, hsa-mir-30c appears to have the highest chance to target NOV as a unique site is predicted for hsa-mir-30c that is not shared by the other mir-30 family members (Additional file 2: Figure S2). Based on ontological terms associated with NOV, and the observed reduction of NOV protein and mRNA levels in response to increased hsa-mir-30c levels, we suggest that NOV is involved in the regulation of MDA-MB-231 invasiveness.

### hsa-mir-30c regulation of NOV and invasion is independent of RUNX2

**Figure 4:**
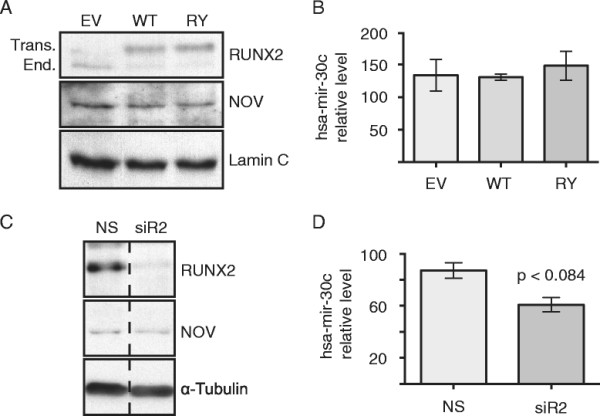
**RUNX2 does not significantly regulate the expression levels of either hsa-mir-30c or NOV. (A, B)** Detection of NOV and hsa-mir-30c levels in MDA-MB-231 stably expressing empty vector (EV), wild-type Runx2 (WT), or R398A/Y428A mutant Runx2 (RY). **(A)** Representative Western blots of MDA-MB-231 stable cell lysates. Top blot: RUNX2 (top band: transgenic murine Runx2, lower band: endogenous human RUNX2). Middle blot: NOV. Lower blot: Lamin C. **(B)** qPCR detection for hsa-mir-30c in consecutive (N = 2) passages of stable MDA-MB-231 cells normalized to U6 snRNA. **(C, D)** Detection of NOV and hsa-mir-30c following 48 h of transient transfection of non-targeting siRNA (NS) and RUNX2 siRNA (siR2). **(C)** Representative Western blots of MDA-MB-231 lysates following 48 h of siRNA transfection. Vertical dashed line indicates that the image of the blot was cut for figure. Top blot: RUNX2. Middle blot: NOV. Lower Blot: α-Tubulin. **(D)** qPCR detection of hsa-mir-30c levels of two technical replicates each of four biological replicates following 48 h transfection of siRNA. p-value from Student’s t-test with Welch correction: approaching statistical significance (*P*-value = 0.0835). **(B, D)** Bars equal mean, error bars equal SEM.

Numerous factors have been implicated in the progression of breast cancer metastasis, and among these is RUNX2, a bone-lineage transcription factor that promotes the invasive phenotype of MDA-MB-231 cells [53]–[55]. Because mmu-mir-30c is known to target Runx2 [13], we addressed whether there is regulatory cross talk between RUNX2, hsa-mir-30c, and NOV in breast cancer cells. RUNX2 levels were modulated in MDA-MB-231 by several approaches: overexpression, siRNA knockdown and by examining a functionally-deficient mutant form of *Mus musculus* Runx2 (R398A/Y428A), which inhibits the invasiveness of MDA-MB-231 [54]–[56] (Figure 4). We observed that constitutive overexpression of Runx2 or the R398A/Y428A-mutant form of Runx2 in MDA-MB-231 cell lines (see Methods) did not alter either the protein levels of NOV (Figure 4A) or the levels of hsa-mir-30c (Figure 4B), which were measured by Western blot and Real Time qPCR, respectively. Knockdown of RUNX2 via siRNA has also been previously shown to inhibit the invasiveness of MDA-MB-231 cells [53]. To further investigate whether RUNX2 can regulate the hsa-mir-30c/NOV pathway, endogenous RUNX2 levels in MDA-MB-231 cells were reduced via siRNA. We observed that NOV protein levels were not affected by RUNX2 knockdown (Figure 4C), and that hsa-mir-30c levels, while slightly reduced, were not statistically significantly changed by RUNX2 siRNA (Figure 4D). These results demonstrate that the hsa-mir-30c/NOV-mediated regulation of the invasiveness of MDA-MB-231 cells is occurring through a RUNX2-independent pathway.

### NOV inhibits the invasiveness of MDA-MB-231 cells

**Figure 5:**
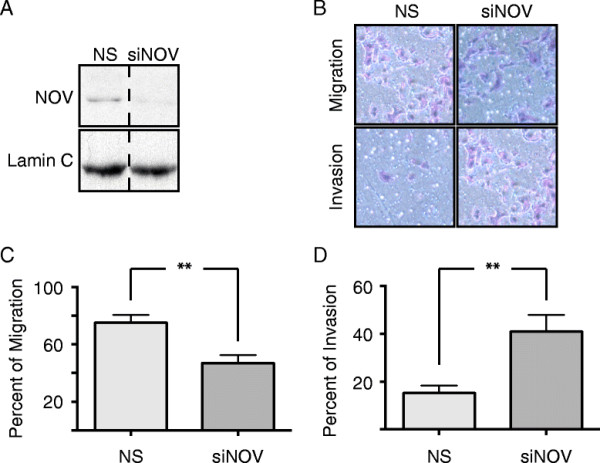
**NOV inhibits the invasiveness of MDA-MB-231 cells. (A)** Representative Western blot for NOV (upper blot) and tubulin (lower blot) 48 h post-transfection with siRNA. Vertical dashed line indicates where image of gel was cut for figure. **(B)** Representative image of HEMA-3 stained cells, which migrated through either the control inserts (upper row) or Matrigel inserts (lower row) after 48 h of transfection with siRNA. **(C)** Quantification of 4 technical replicates of 2 biological replicates measuring the percent of cells that migrated through the control inserts (100 % being the number of cells loaded into the inserts). **(D)** Quantification of 4 technical replicates of 2 biological replicates measuring the percent of cells that invaded through the Matrigel normalized by the number of cells migrated through the control inserts. **(A-D)** NS = Non-silencing siRNA, siNOV = NOV siRNA. **(C, D)** Bars equal mean, error bars equal SEM. ** = Wilcoxon Rank Sum p-value < 0.01.

**Figure 6:**
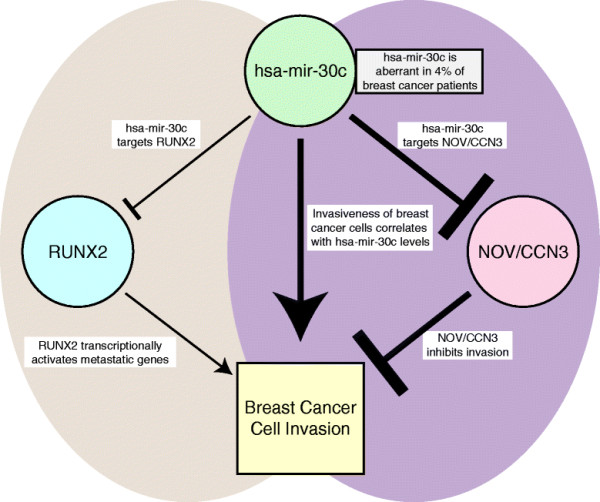
**Model of impact of hsa-mir-30c on breast cancer cell invasion.** Left side: hsa-mir-30c targets and inhibits RUNX2 levels (data not shown and [13]); RUNX2 expressed in metastatic breast cancer cells promotes breast cancer cell invasion [14]. Right side: hsa-mir-30c targets and inhibits NOV/CCN3 levels (Figure 3); NOV/CCN3 inhibits breast cancer cell invasion (Figure 5). Thus the down regulation of NOV/CCN by miR30c contributes to invasive properties. The hsa-mir-30c-RUNX2 pathway is independent of the hsa-mir-30c-NOV/CCN3 pathway (Figure 4). This model of two factors RUNX2 and NOV, each with opposing roles in the invasiveness of metastatic breast cancer cells, are targets of a miR-30c regulatory circuit that reflects the hsa-mir-30c is likely to have many targets that potentially contribute to different properties of breast cancer cells.

To determine the extent to which hsa-mir-30c targeting and reduction of NOV was involved in the increased invasion of MDA-MB-231 cells following transfection of hsa-mir-30c, we transfected MDA-MB-231 cells with NOV-specific siRNA to reduce NOV protein levels without directly affecting other targets of hsa-mir-30c (Figure 5A). We postulate if hsa-mir-30c-mediated down-regulation of NOV levels is a key contributor to the invasiveness of MDA-MB-231 cells, we should therefore observe that siRNA-mediated knockdown of NOV results in an invasive phenotype similar to overexpression of hsa-mir-30c. The levels of NOV were significantly reduced by the siRNA (Figure 5A), and using Matrigel invasion assay, we observed an increase in the number of MDA-MB-231 that invaded through the Matrigel (Figure 5B-D). MDA-MB-231 cells lacking NOV were significantly more invasive (Figure 5D), yet were less migratory (Figure 5C). While it is often observed that trends in invasion and migration are positively correlated, we observed less migration coupled with increased invasion in the absence of NOV. These results suggest that in MDA-MB-231 cells, NOV has distinct regulatory roles for the pathways governing invasion and migration. Taken together, our findings support the conclusion that the targeting of NOV by hsa-mir-30c is an important factor in the invasive phenotype evidenced by the MDA-MB-231 cells as illustrated in Figure 6.

## Discussion

Here we identify a novel pathway by which hsa-mir-30c promotes the invasiveness of the MDA-MB-231 cell line through targeting of NOV. The specificity of NOV’s involvement in the invasive phenotype observed by several experimental approaches including the knockdown by an siRNA targeting NOV, which resulted in significant increases in the invasiveness of MDA-MB-231, consistent with the high levels of hsa-mir-30c, and NOV cellular protein levels in these cells. These results demonstrate a novel pathway by which a miRNA (hsa-mir-30c) promotes the invasive phenotype of metastatic breast cancer cells.

The large mir-30 family shares a conserved seed sequence. However, our *in silico* research suggests that seed sequence differences may give rise to selectivity in targeting aggressive compared to modestly invading cells among mir-30 members. It is appreciated from the literature that the miR-30 family has numerous targets, and others have been identified in breast [57]–[59] and other cancers [30],[60]–[64]. For example, a recent study showed that has-mir-30a marker targets the 3’-UTR of Vimentin (VIM) which was observed to cause reductions in both VIM protein levels and invasiveness of MDA-MB-231 cells [63], and here we demonstrate that miR-30c also promotes cellular invasion by targeting NOV. It is also quite interesting that these mir-30 miRNAs appear on many chromosomes rather than co-regulated in a cluster, and are involved in the regulation of a myriad of pathways such as tumor suppression (p53) [63], apoptosis (BCL) [64], and epithelial to mesenchymal transition (VIM) [59]. Individual members of the mir-30 family have been implicated in both tumor suppression and oncogenesis; it is therefore difficult to define the family as a “tumor suppressive” or “oncogenic”. This ambiguity makes studying the functions of the mir30 family members on a case-by-case basis critical for understanding the basis of post-transcriptional molecular mechanisms of disease. The relative levels of the mir-30 family members and their temporal expression may play a critical role in disease progression.

Also, to be considered is the context in which our experiments were conducted. We investigated the functions of hsa-mir-30c in MCF-7, a less invasive breast cancer cell line and the more invasive MDA-MB-231 cells, which is a cell model of advanced metastatic breast cancer [65]. We find a positive in correlation in levels of hsa-mir-30c and invasion properties *in vitro* assays and high expression of hsa-mir-30c has been observed in MDA-MB-231cells, as well as in other cellular models of breast cancer. However detection of hsa-mir-30c did not appear to be predictive of breast cancer subtypes [66]. Our cell-based observations are similar to observations made in *in vivo* studies of miRNA expression patterns in breast cancer patient samples in which hsa-mir-30c expression was not significantly associated with any clinical or pathological features [12],[67]–[70]. However, elevated expression of hsa-mir-30c was observed to be significantly associated with estrogen receptor (ER) positive breast cancers that were beneficially responsive to tamoxifen treatment [58]. In a separate study, ER-positive breast cancer patients who relapsed after tamoxifen treatment had significantly increased levels of NOV [49], which further underscores the inverse relationship between NOV and hsa-mir-30c in breast cancer, as well as in our observations of low hsa-mir-30c in the ER positive cell line (MCF-7) and higher levels in the MDA-MB-231 cells which are ER negative.. Thus, we have observed that hsa-mir-30c was important for the invasive properties which is discordant with the *in vivo* observations that hsa-mir-30c expression was not positively associated with metastatic disease, but rather, with positive outcome in response to hormone therapy. We therefore suggest that the cohort of potential mRNA targets available to hsa-mir-30c in patients may be quite different from those in MDA-MB-231 cells, which could explain the differences between *in vivo* tumors with a heterogenous tumor cell population and individual cell lines. The significance of our findings is that there are likely to be tumor cells during progression of breast cancer *in vivo* that could benefit from targeted hsa-mir-30c or NOV therapy in reducing invasion and later stage metastasis.

We have demonstrated that the functional consequence of increased invasiveness of MDA-MB-231 cells following the reduction of NOV through either hsa-mir-30c or NOV-siRNA is directly sensitive to NOV levels. NOV appears to play a context-sensitive role in oncogenesis/tumor suppression [71]. In several cancers that develop from mesenchymal tissues, NOV has been shown to promote tumor growth and metastasis [72]–[74]. However, in the context of brain cancer, NOV appears to inhibit disease progression [53],[75]–[77], but in the context of alveolar rhabdmyosarcoma NOV/CCN3 levels are increased and contribute to survival of oppressive behaviors of the cancerous cells promotes motility. While there are conflicting observations of NOV functions in breast cancer cell lines [28],[49], NOV protein levels in human tissue samples of breast cancer were observed to be negatively correlated with late-stage and metastatic disease [50]. These histological observations suggest that in breast cancer, NOV functions to inhibit disease progression.

## Conclusions

We have demonstrated that hsa-mir-30c-mediated inhibition of NOV levels promotes the invasive phenotype of MDA-MB-231 cells. We find that lower levels of the canonical hsa-mir-30c are correlated with modest invasion of MCF-7 compared to the aggressive invasion of MDA-MD-231 cells, which express high levels of hsa-mir-30c. The mechanism we have uncovered related to hsa-mir-30c is that that the functional consequence of increased invasiveness of MDA-MB-231 cells following the targeting and reduction of NOV through either hsa-mir-30c or NOV-siRNA and that the invasive properties of the metastatic breast cancer cell line MDA-MB-231i are directly sensitive to NOV levels. NOV/CCN3 expression in patients is known to inversely correlate with advanced breast cancer and metastasis. Thus, a key function of NOV is the inhibition of the invasiveness of metastatic breast cancer cells.

## Methods

### Cell Properties

#### Cell lines

MCF-7 cells were grown in DMEM in media and MDA-MD-231 cells in alpha MEM and maintained as previously described [53]. These cells have been maintained in our laboratory and used in seven publications since 2005. In 2012 the cells were revalidated by satellite phenotyping. These cell lines were grown and maintained as previously described [53]. Constructs and stable cell lines were generated as previously described [56]. MDA-MB-231 cells which were lentivirus-transduced stable cell lines to express either empty vector, wild-type *Mus musculus* Runx2 a subnuclear-targeting-deficient mutant form of *Mus musculus* Runx2 (R398A/Y428A), which inhibits the invasiveness of MDA-MB-231 [54]–[56] were used in these studies.

### RNA isolation

RNA was isolated using Qiagen miRNAeasy Mini Kit (217004) following the manufacturer’s recommended protocol with optional in-column DNAse I digestion of genomic DNA (Qiagen RNase-Free DNase Set 79254).

### miRNA amplification and detection

Complimentary miRNA-specific cDNA was amplified and detected using Applied Biosystems TaqMan MicroRNA Assays for hsa-mir-30c (#4427975) hsa-mir-30c-2* (#4427975) and RNU6B (#4427975).

### cDNA amplification and detection

cDNA was amplified from equal quantities of total cellular RNA for each treatment or cell line. cDNA was amplified using the Invitrogen SuperScript First-Strand Synthesis System for RT-PCR (#11904-018) according to the manufacturer’s protocol. Reactions were volumetrically diluted, and reaction products were used as templates for Real Time qPCR using Bio-Rad iQ SYBR Green Supermix (#170-8880).

### cDNA qPCR primers

Real Time qPCR primers were designed using FoxPrimer (www.foxprimer.org) and validated for efficiency by standard curve using cDNA amplified from untreated MDA-MB-231 cells.

### Protein isolation and Western blotting

Cells grown on tissue culture plates were placed directly on ice, and washed twice with PBS supplemented with Roche cOmplete, EDTA-free Protease Inhibitor Cocktail (#11873580001) and 25 μM MG132 (Calbiochem (EMD Millipore) CAS 133407-82-6). Cells were scraped into screw-top microcentrifuge tubes, gently spun down to pellet cells and excess PBS was aspirated and discarded. Cells were snap-frozen in liquid nitrogen. Protein lysates were prepared by the addition of RIPA buffer (50 mM Tris pH 7.4, 150 mM NaCl, 2 mM EDTA, 1% v/v NP-40, 0.1% w/v SDS, 1× Roche cOmplete, EDTA-free Protease Inhibitor Cocktail and 25 μM MG132) and placing tubes on a 100 °C heat block for 10 min. Protein lysates were quantified using Pierce BCA Protein Assay Kit (#23225) according to manufacturer’s instructions. Fifty μg protein per sample was loaded onto an SDS-PAGE gel. SDS-PAGE was performed as described [53]. Briefly, lysates were run through an 8.5% acrylamide gel, and then transferred to a PVDF Transfer Membrane (Thermo Scientific #88518).

Membranes were blocked with 5% (w/v) milk (BioRad #170-6404XTU) in PBS and then subjected to immunodetection using the following primary antibodies and dilution factors in 1% (w/v) milk in PBS: NOV (Santa Cruz Biotechnology H-71 sc-50304 1:1000), Lamin A/C (Santa Cruz Biotechnology N-18 sc-6215 1:5000), α-Tubulin (Santa Cruz Biotechnology H-300 sc-5546 1:2000), RUNX2 (mouse hybridoma clone 8G5 1:1000) [53], FOXG1 (Santa Cruz Biotechnology N-15 sc-1858), SATB2 (Santa Cruz Biotechnology H-118 sc-98677), SMAD2 (Santa Cruz Biotechnology S-20 sc-6200), MTSS1 (Santa Cruz M7-P3A7 sc-101390), WWP1 (Santa Cruz Biotechnology N-20 sc-11892), RUNX1 (Active Motif #39000), Cdk2 (Santa Cruz Biotechnology M2 sc-163). Secondary antibodies used were from Santa Cruz Biotechnology and were diluted 1:5000 in 1% (w/v) milk in PBS: donkey anti-goat IgG-HRP (sc-2020), goat anti-mouse IgG-HRP (sc-2005), and goat anti-rabbit IgG-HRP (sc-2004). After incubation with primary and secondary antibodies, the membranes were washed three times for 30 min each with 0.1% (v/v) Tween-20 in PBS. HRP reaction was achieved by one minute incubation with Perkin Elmer Western Lightning ECL (NEL102001EA). Membranes were exposed to Kodak BioMax Light File for Chemiluminescent Imaging (#868-9358) in serial exposure times to empirically determine the exposure time at which the signal was most linear.

### Matrigel invasion and migration assays

Proliferating MDA-MB-231 cells were trypsinized and counted using Cellometer Auto T4 Cell Counter. A cell suspension of 100,000 cells/mL in growth medium was prepared and 100 μL of the suspension was loaded into each BD Matrigel 24-well 8.0 μm PET Membrane Invasion Chamber (#354483). Matrigel coated plates, and control insert plates had 500 μL NIH3T3-conditioned medium loaded in the bottom as the chemoattractant. Plates and chemoattractant medium were incubated at 37 °C for 3–4 h prior to loading MDA-MB-231 cells. Cells were incubated for 16 h at 37°C in 5% CO_2_ and then fixed and stained using the Fisher HealthCare PROTOCOL Hema 3 Manual Staining System (#22-122-911) according to the manufacturer’s instructions. Cotton swabs were used to eliminate cells which did not migrate/invade as well as Matrigel. Cells were counted using an inverted light microscope. To control for proliferation effects, rates of cellular invasion through Matrigel were normalized by rates of cellular migration through control plastic-only insert wells.

### Transient transfection

Proliferating MDA-MB-231 cells were transfected with 50nM of siRNA/miRNA using Oligofectamine (Invitrogen #12252-011) according to the Oligofectamine protocol.

### siRNAs

Dharmacon SMARTpool: ON-TARGETplus RUNX2 siRNA (L-012665-00-0005); Dharmacon SMARTpool: ON-TARGETplus NOV siRNA (L-010527-00-0005); Dharmacon ON-TARGETplus Non-targeting Pool (D-001810-10-05).

### miRNAs and anti-miRNAs

Dharmacon miRIDIAN microRNA hsa-mir-30c-1 mimic (C-300542-03-0005); Dharmacon miRIDIAN microRNA hsa-mir-30c-1* mimic (C-301199-01-0005); Dharmacon miRIDIAN microRNA hsa-mir-30c-1 haripin inhibitor (IH-300542-07-0005); Dharmacon miRIDIAN microRNA Mimic Negative Control #1 (CN-001000-01-05); Dharmacon miRIDIAN microRNA Hairpin Inhibitor Negative Control #1 (IN-001005-01-05).

### Screen for hsa-mir-30c targets

The top 300 targets of hsa-mir-30c based on mirSVR were downloaded from www.microrna.org in January 2011. Gene symbols were used to access gene ontology (GO) terms from DAVID (http://david.abcc.ncifcrf.gov) and gene reference into function (GeneRIF) from NCBI (http://www.ncbi.nlm.nih.gov/gene/about-generif). Genes whose GO terms or GeneRIFs were associated with invasion, migration, extracellular matrix, or transcription factors were selected and qPCR primers were designed. After 48 h of transfection, RNA was isolated, cDNA was amplified and Real Time qPCR was carried out to detect the relative levels of mRNAs following transfection with hsa-mir-30c.

## Abbreviations

3’-UTR: Three prime untranslated region

ADAM22: Homo sapiens ADAM metallopeptidase domain 22

BCL: Homo sapiens B-cell lymphoma 2

BMP-2: Homo sapiens bone morphogenetic protein 2

BUB3: Homo sapiens BUB3 mitotic checkpoint protein

CCN: Family of extracellular matrix proteins, which includes NOV

CELSR3: Homo sapiens cadherin EGF LAG seven-pass G-type receptor 3

DYNLT3: Homo sapiens dynein, light chain, Tctex-type 3

EGF: Homo sapiens epidermal growth factor

ER: Homo sapiens estrogen receptor alpha

GO: Gene ontology

HPRT: Homo sapiens hypoxanthine-guanine phosphoribosyltransferase

MCF-7: Poorly invasive, malignant breast cancer cell line

MDA-MB-231: Metastatic breast cancer cell line

NEDD4: Homo sapiens neural precursor cell expressed, developmentally down-regulated 4, E3 ubiquitin protein ligase

NOV/CCN3: Homo sapiens nephroblastoma overexpressed

PTEN: Homo sapiens phosphatase and tensin homolog

PTPN3: Homo sapiens protein tyrosine phosphatase, non-receptor type 3

R398A: Arginine 398 mutated to alanine

RNA-seq: Ribonucleic acid sequencing

RUNX2: Homo sapiens runt-related transcription factor 2

Runx2: Mus musculus runt-related transcription factor 2

SRC-1: Homo sapiens V-Src avian sarcoma (Schmidt-Ruppin A-2) viral oncogene homolog

TBFβ1: Homo sapiens transforming growth factor, beta 1

TWF1: Homo sapiens twinfilin actin-binding protein 1

VIM: Homo sapiens vimentin

Y428A: Tyrosine 428 mutated to alanine

hsa-mir-30c: Homo sapiens miRNA 30c

hsa-mir-30c-3p: Alternative, 3’-form of hsa-mir-30c

hsa-mir-30c-5p: Canonical, 5’-form of hsa-mir-30c

mRNA: Messenger ribonucleic acid

metasta-miR: An miRNA that promotes cancer cell metastasis

miRNA: Small non-coding ribonucleic acid

mirSVR: A score derived from a machine learning method for ranking miRNA target sites

mmu-mir-30c: Mus musculus miRNA 30c

onco-miR: An miRNA that has oncogenic functions

p-Smad1: Homo sapiens SMAD family member 1, phosphorylated

p53: Homo sapiens tumor suppressor protein 53

pre-miRNA: immature transcript of an miRNA

qPCR: Quantitative polymerase chain reaction

siRNA: Short interfering ribonucleic acid

## Competing interests

The authors declare that they have no competing interests.

## Authors’ contributions

qPCR for miRNAs performed by JRD and HT. qPCR for RUNX2 and NOV performed and analyzed by JRD. siRNA and miRNA transfections performed by JRD. Invasion assays performed by JRD and HT. Western blotting performed by JRD. MDA-MB-231 cells stably overexpressing Runx2 and Runx2-RY engineered by DH. Initial cancer-centric screens for hsa-mir-30c targets in MDA-MB-231 cells performed by YH and JP. Ontological and qPCR screen was designed and executed by JRD. Study was executed under the guidance of JBL, AJVW, JLS, GSS, and JP, who contributed to the writing of this manuscript. All authors read and approved the final manuscript.

## Additional files

## Supplementary Material

Additional file 1: Figure S1. Western blot screening of potential cancer-related targets of hsa-mir-30c. (A and B) Western blots showing protein levels of FOXG1, SATB2, SMAD2, MTSS1, WWP1, RUNX1, and CDK2 in whole cell lysates from MDA-MB-231 cells following 36 hours of transient transfection with either mock (M), non-targeting miRNA (NT), or hsa-mir-30c (30c). Click here for file

Additional file 2: Figure S2. Alignment of mir-30 family members on the NOV 3’-UTR. For each of the predicted sites of targeting by hsa-mir-30c on the NOV 3’-UTR (A-C), if an alignment is possible, the alignment of each mir-30 member is presented. Alignment of hsa-mir-30 family members (top sequences) with the 3’-UTR of NOV (bottom sequences); the 5’-positions within the NOV 3’-UTR are relative to the 5’-start of the 3’-UTR for each of the three predicted targeting sites. Target scores are provided by mirSVR. Uppercase letters linked with a “|” character indicates a perfect match, while uppercase letters linked with a “:” indicate a wobble pair. Click here for file
