# Supplementary figures and images for: hsa-mir-30c promotes the invasive phenotype of metastatic breast cancer cells by targeting NOV/CCN3
Source: Cancer Cell Int. 2014 Aug 2;14:73. doi: 10.1186/s12935-014-0073-0 (PMC4129468; doi:10.1186/s12935-014-0073-0)

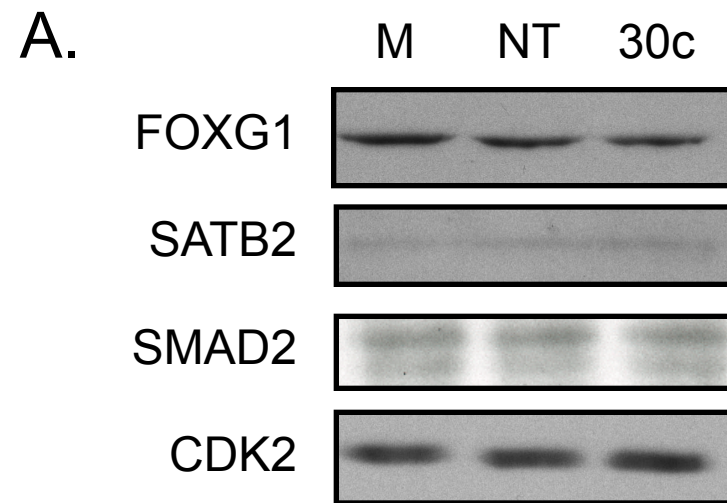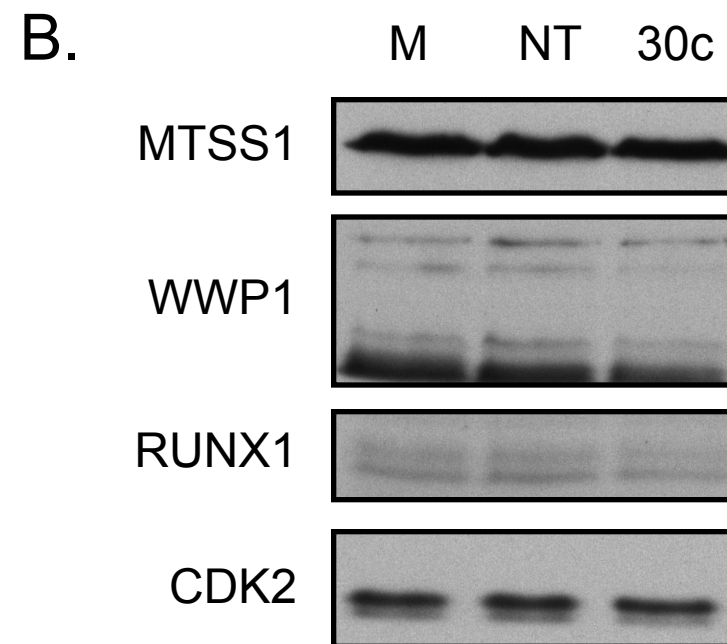

Supplement: Additional file 1: Figure S1. — Western blot screening of potential cancer-related targets of hsa-mir-30c. (A and B) Western blots showing protein levels of FOXG1, SATB2, SMAD2, MTSS1, WWP1, RUNX1, and CDK2 in whole cell lysates from MDA-MB-231 cells following 36 hours of transient transfection with either mock (M), non-targeting miRNA (NT), or hsa-mir-30c (30c). [file s12935-014-0073-0-S1.pdf]
