# Supplementary material for: hsa-mir-30c promotes the invasive phenotype of metastatic breast cancer cells by targeting NOV/CCN3
Source: Cancer Cell Int. 2014 Aug 2;14:73. doi: 10.1186/s12935-014-0073-0 (PMC4129468; doi:10.1186/s12935-014-0073-0)
Supplement: Additional file 2: Figure S2. — Alignment of mir-30 family members on the NOV 3’-UTR. For each of the predicted sites of targeting by hsa-mir-30c on the NOV 3’-UTR (A-C), if an alignment is possible, the alignment of each mir-30 member is presented. Alignment of hsa-mir-30 family members (top sequences) with the 3’-UTR of NOV (bottom sequences); the 5’-positions within the NOV 3’-UTR are relative to the 5’-start of the 3’-UTR for each of the three predicted targeting sites. Target scores are provided by mirSVR. Uppercase letters linked with a “|” character indicates a perfect match, while uppercase letters linked with a “:” indicate a wobble pair. [file s12935-014-0073-0-S2.pdf]

A.

```
                mirSVR Score: -1.0927
      3'   cgacucucACAUCCUACAAA-UGu        5' hsa-mir-30c
                   |||||    ||||| ||
 290: 5'   auuuacuuUGUAGACUGUUUCACa        3' NOV
```

B.

```
                mirSVR Score: -1.0446
      3'   gaAGGUCA--GCUCCUACAAAUGu        5' hsa-mir-30a
             | ||||   :||   ||||||||
 591: 5'   ucUACAGUAAUGA-AAUGUUUACu        3' NOV

                mirSVR Score: -1.0419
      3'   ucgacUCA-CAUCCUACAAAUGu         5' hsa-mir-30b
                |||  |:     ||||||||
 591: 5'   ucuacAGUAAUGAAAUGUUUACu         3' NOV

                mirSVR Score: -1.0419
      3'   cgacUCUCA-CAUCCUACAAAUGu        5' hsa-mir-30c
               |  |||  |:    ||||||||
 590: 5'   aucuACAGUAAUGAAAUGUUUACa        3' NOV

                mirSVR Score: -1.0446
      3'   gaAGGUCA-GCCCCUACAAAUGu         5' hsa-mir-30d
             | ||||   |    ||||||||
 591: 5'   ucUACAGUAAUGAAAUGUUUACu         3' NOV

                mirSVR Score: -1.0392
      3'   gaAGGUCAGUUC-CUACAAAUGu         5' hsa-mir-30e
             | |||| | |   ||||||||
 591: 5'   ucUACAGUAAUGAAAUGUUUACu         3' NOV
```

C.

```
                mirSVR Score: -0.9748
      3'   gaaggUCAGCU----CCUACAAAUGu      5' hsa-mir-30a
                |||:||     |  |||||||
1248: 5'   auaaaAGUUGAACAUUGUUGUUUACu      3' NOV

                mirSVR Score: -0.9779
      3'   ucgacucacaucCUACAAAUGu          5' hsa-mir-30b
                       |  |||||||
1252: 5'   aaguugaacauuGUUGUUUACu          3' NOV

                mirSVR Score: -0.9779
      3'   cgacucucacaucCUACAAAUGu         5' hsa-mir-30c
                        |  |||||||
1251: 5'   aaaguugaacauuGUUGUUUACu         3' NOV

                mirSVR Score: -0.9748
      3'   gaaggucagcccCUACAAAUGu          5' hsa-mir-30d
                       |  |||||||
1252: 5'   aaguugaacauuGUUGUUUACa          3' NOV

                mirSVR Score: -0.9748
      3'   gaaggucaGUUCCUACAAAUGu          5' hsa-mir-30e
                   ||  ||  ||||||||
1252: 5'   aaguugaaCAUUGUUGUUUACu          3' NOV
```
